# Assessing Nerve Conduction Velocity as a Diagnostic and Prognostic Indicator in Leprosy Patients: A Cross-Sectional Study

**DOI:** 10.7759/cureus.58294

**Published:** 2024-04-15

**Authors:** Milind A Nisargandha, Shweta D Parwe, Nitin R Lade, Jabir Padathpeedika Khalid, Vaishali V Kuchewar

**Affiliations:** 1 Physiology, Saveetha Medical College and Hospital, Saveetha Institute of Medical and Technical Sciences, Saveetha University, Chennai, IND; 2 Panchakarma, Mahatma Gandhi Ayurved College Hospital and Research Centre, Datta Meghe Institute of Higher Education and Research (DMIHER), Wardha, IND; 3 Dermatology, Venereology and Leprosy, All Indian Institute of Medical Sciences, Nagpur, IND; 4 Kayachikitsa, Mahatma Gandhi Ayurved College Hospital and Research Centre, Datta Meghe Institute of Higher Education and Research (DMIHER), Wardha, IND

**Keywords:** nerve impairment, nerve conduction velocity (ncvs), neuropathy, mycobacterium leprae, hansen’s disease

## Abstract

Introduction

Hansen's disease is a condition in which patients develop peripheral neuropathy. In 1873, G. H. A. Hansen discovered Mycobacterium leprae, the causative agent of leprosy, a chronic infectious disease. These bacteria influence the peripheral nerves, which is likely to cause neuropathy. Sensory nerve conduction studies were performed in leprosy patients on the upper limb nerves of 30 patients in the rural area of the Wardha district in the Indian population.

Methods

In this study, we recruited 30 leprosy patients from the Department of Dermatology and A.V.B.R. Hospital, Sawangi Wardha. The patient's nerve conduction velocity (NCV) tests were carried out in the Department of Physiology at J. N. Medical College, Wardha. NCVs were obtained during these three years, beginning in 2009, while performing sensory nerve conduction velocity (SNCV) and motor nerve conduction velocity (MNCV). The latency, amplitude, and NCV parameters were recorded, and the data collection period ended in 2011. In this study, we measured both MNCV and SNCV.

Results

In our study, impairment of conductional velocity was observed. In leprosy patients, the MNCV values of latency, amplitude, and conductional velocity were 6.61, 3.89, and 46.92 m/s, respectively, whereas the SNCV values were 3.005, 25.17, and 38 m/s, respectively. Based on the results, it appears that the maximal sensory nerve involvement was recorded at 38 m/s conductional velocity. In NCVs, increased latency and decreased conductional velocity were found across the study.

Conclusion

It was concluded that nerve conduction studies are one of the non-invasive techniques for early diagnosis and management of leprosy. This study should be repeated with a larger sample size and should be multicentric.

## Introduction

*Mycobacterium leprae* is a type of bacteria that causes leprosy, a chronic infectious disease [1]. The number of leprosy patients is approximately 10.6 million worldwide. About 62% were observed in Asia alone. Approximately 250,000 new leprosy cases are registered every year, and about two million people suffer from leprosy-related disabilities [2]. Leprosy predominately affects the skin, peripheral nerves, and mucous membranes. The signs and symptoms of leprosy can be broken down into three groups: peripheral neuritis [3], the immune system's response to bacilli, and the way bacteria get into the body.

*M. leprae* is an acid-fast bacterium that primarily affects the skin, upper respiratory tract, and nerves. This infection results in leprosy. As a chronic condition, it mainly affects different sites like mucus membranes, eyes, and testes. It also causes all of *M. leprae*'s clinical symptoms [4,5], which are mostly caused by the organism attaching to skin cells like macrophages, keratinocytes, and histiocytes [6,7]. In the peripheral nerves, *M. leprae* mainly occurs in the Schwann cells [4]. When the antigens of *M. leprae* are recognized, keratinocytes are known to release an important antimicrobial peptide into the body [6]. In a host cell, *M. leprae* interacts with lipid metabolism for intracellular bacterial survival [7].

Even in highly endemic places, most people successfully resist *M. leprae* infection. It is now estimated that around 200 people have been infected with *M. leprae*. Each open case develops slowly, multiplies, and causes damage to the skin as this involvement is detected [8]. The consistency of the host's immune response determines the disease's various clinical manifestations. The most effective steps in avoiding deformity and impairment are the early diagnosis of leprosy and multidrug therapy treatment [9]. The bacilli present in the skin cause the disease's dermatological manifestations. At the same time, nerve infection causes axonal demyelination, resulting in sensory deprivation and its deformity implications.

Individuals diagnosed with leprosy who have less than five lesions with leprosy are classified as paucibacillary (PB), whereas those who have more than five are classified as multibacillary (MB). Patients can experience numbness, muscle fatigue, or paralysis due to thickened peripheral nerves [10]. Untreated patient droplets can transmit the MB infection through the mouth and nose if patients come into close contact. When these people cough or sneeze, respiratory droplets may be released that contain the persistent infection. The World Health Organization (WHO) established a new strategy in April 2019 to hasten the reduction of the leprosy disease burden and achieve a leprosy-free world by 2030 in response to the threat that leprosy patients pose to human health [11]. India, Brazil, and Indonesia had the maximum number of cases reported in 2016. This may lead to a global leprosy burden in middle-income countries predominately.

For a long time, the government emphasized leprosy's public policies based on dermatological signs rather than neurological symptoms [12]. Medical reports indicate this disease has many difficult human symptoms to diagnose [13]. Experienced medical professionals can also speed up the diagnosis. Hence, it has been made mandatory to use electrodiagnosis for nerve involvement in leprosy patients. Peripheral nerve lesions are the most common cause of disability in leprosy. Neuropathy can easily be detected through electrophysiological tests to diagnose diseases like leprosy.

The degenerative changes can be correlated with the infection of the sensory nerves [14], an important event in the natural history of leprosy. Because of their inflammatory impact on peripheral nerves, leprosy symptoms remain the main contributor to sensory loss and dysfunction once the infection is established.

Peripheral nerve injuries affect people of all ages. Damage to the nerves can happen before, during, or after therapy. Some people do not develop nerve damage, while others develop anesthesia in their hands and feet, putting them at risk of neuropathic injury. People with weak or paralyzed small muscles in their hands, feet, or eyes are at risk of contractures and deformities. In patients with leprosy, repeated injury to weak, anesthetized limbs results in losing fingers and toes [15].

This disease has decreased significantly in the last few decades using multidrug and anti-inflammatory therapies. But still, early detection of leprosy will avoid deformity. Hence, the study was planned with the aim of detecting nerve involvement at an early stage in leprosy patients.

## Materials and methods

Ethical considerations

After careful consideration and gatekeeper consent, the Datta Meghe Institute of Medical Sciences institutional ethical clearance committee approved the study. ICE DMIMSU (DU) Wardha, Maharashtra, India, provided the presentation in front of the I.E.C. (Letter and Reference No. DMIMS (DU)/IEC/2009-10/202). Written informed consent was obtained for the study from all the participants. Anonymity and confidentiality were explained to them before the study began, and the participants voluntarily signed the consent forms. Informed consent included the fact that the participants could withdraw their names from the study at any point if they no longer felt comfortable continuing.

Study design

This was an observational cross-sectional study that was carried out in the Department of Physiology, J.N. Medical College, and Dermatology Department, A.V.B.R. Hospital Sawangi (Meghe) Wardha, over a three-year period (2009-2011) on leprosy patients.

Selection of patients

The present study was conducted on 30 leprosy patients diagnosed in the past one year with PB and MB cases. The patients investigated in the Department of Dermatology were selected based on routine clinical examinations and leprosy symptoms. Nerve conduction study was carried out in the Neurophysiology Lab, Department of Physiology, J.N.M.C. Written informed consent was obtained from the patients in this study after obtaining their history of symptoms regarding the onset of leprosy. A routine clinical examination and blood investigation (complete blood count) were done.

The World Health Organization (WHO) International Classification of Diseases (ICD) version 10 under section A30 Leprosy [Hansen disease] [16] *M. leprae* (ICD-10-CM) for leprosy was used to fulfill the selection criteria of participants, using the following inclusion and exclusion criteria.

Inclusion criteria

Patients infected with *M. leprae* ICD-10-CM Code for Leprosy [Hansen's illness] A30, loss of sensation with hypopigmented/reddish skin lesions, PB single-lesion leprosy: one skin lesion, PB leprosy: two to five patches or lesions on the skin, and the MB leprosy: >5 patches or lesions on the skin were included in this study.

Exclusion criteria

The patient suffering from diabetic neuropathy and neurological disorders were excluded from the study. A routine laboratory test was carried out for the HbA1c. 

The variables evaluated for the diagnosis and treatment of leprosy include nerve involvement. In this study, latency, amplitude, and nerve conduction velocity (NCV) were assessed for the diagnosis of leprosy patients who met the current criteria. The symptoms included 1) lack of sensation of the hypopigmented skin patch and 2) thickened peripheral nerve combined with loss of sensibility. A skin biopsy was taken for histopathology investigation to confirm leprosy.

Data Sources/Measurement

Nerve conduction study: All the patients diagnosed with confirmed leprosy were sent for further investigation to the neurophysiological laboratory in the Department of Physiology. The instrument used for the assessment of NCV variables was the RMS-EMGEP Mark II machine for recording the sensory and motor nerve parameters of the upper extremities.

The motor nerve conduction velocity (MNCV) characteristics were measured for distal delay (latency), compound action potential amplitude, and sensory nerve conduction velocity amplitude (SNAP). Similarly, sensory nerve conduction velocity (SNCV) onset latency, amplitude, and sensory nerve conduction velocity were measured for both upper arms. For the motor studies, the filters were set at 2-5 Hz, and the sweep speed was 5 ms for each division. In the sensory analysis, the filters were 20-3 kHz and 2 ms per division. The NCV of MNCV and SNCV recordings was set for 100 s [17].

The motor nerve characteristics were distal latency, compound action potential, and conduction velocity. The wrist and elbow were the stimulation locations for both nerves (median and ulnar) and the recording sites for the motor points of abductor pollicis brevis and abductor digiti minimi. The reference electrode for the median nerve was placed 4 cm distally over the first metacarpophalangeal joint and the ulnar nerve's fifth metacarpophalangeal joint. Sensory nerves, onset latency, sensory nerve action potential (SNAP), and conduction velocity, on the other hand, were recorded. The electrodes were placed on the index or little finger for the median and ulnar nerves in an antidromic examination for sensory nerves. SNAP amplitude was measured from peak to base. Between the stimulation and recording electrodes, the ground electrodes were placed. No bias was identified in this study.

Sample size

The sample size of 30 leprosy patients was calculated by using the software, Epi Info Statistical Software version 7.2 (https://www.cdc.gov/epiinfo/pc.html).

To calculate the sample size, the following factors need to be considered. The level of significance (alpha) is typically set at 0.05. The desired power of the study (1-beta) is typically set at 0.8. The expected effect size is the difference in nerve conduction velocity between the leprosy patients and the healthy individuals. The standard deviation of nerve conduction velocity in the population. Assuming an alpha of 0.05, a power of 0.8, an effect size of 0.5, and a standard deviation of 10 m/s, the sample size can be calculated using the following formula (Equation 1):

n = (Z_alpha/2 + Z_beta)^2^ * (SD^2^)/effect size^2^, (Equation 1)

where Z_alpha/2 is the value from the standard normal distribution corresponding to the level of significance (1.96 for alpha = 0.05), Z_beta is the value from the standard normal distribution corresponding to the desired power (0.84 for power = 0.8), SD is the standard deviation of nerve conduction velocity, and effect size is the expected difference in nerve conduction velocity.

n = (1.96 + 0.84)^2^ * (10^2^) / 0.5^2^ = 30.

Thus, the sample size required for this study would be 30 leprosy patients.

Statistical methods

The data were analyzed using Microsoft Excel version 11 for the analysis. The data are represented by the average values depicted in the result.

## Results

**Figure 1:**
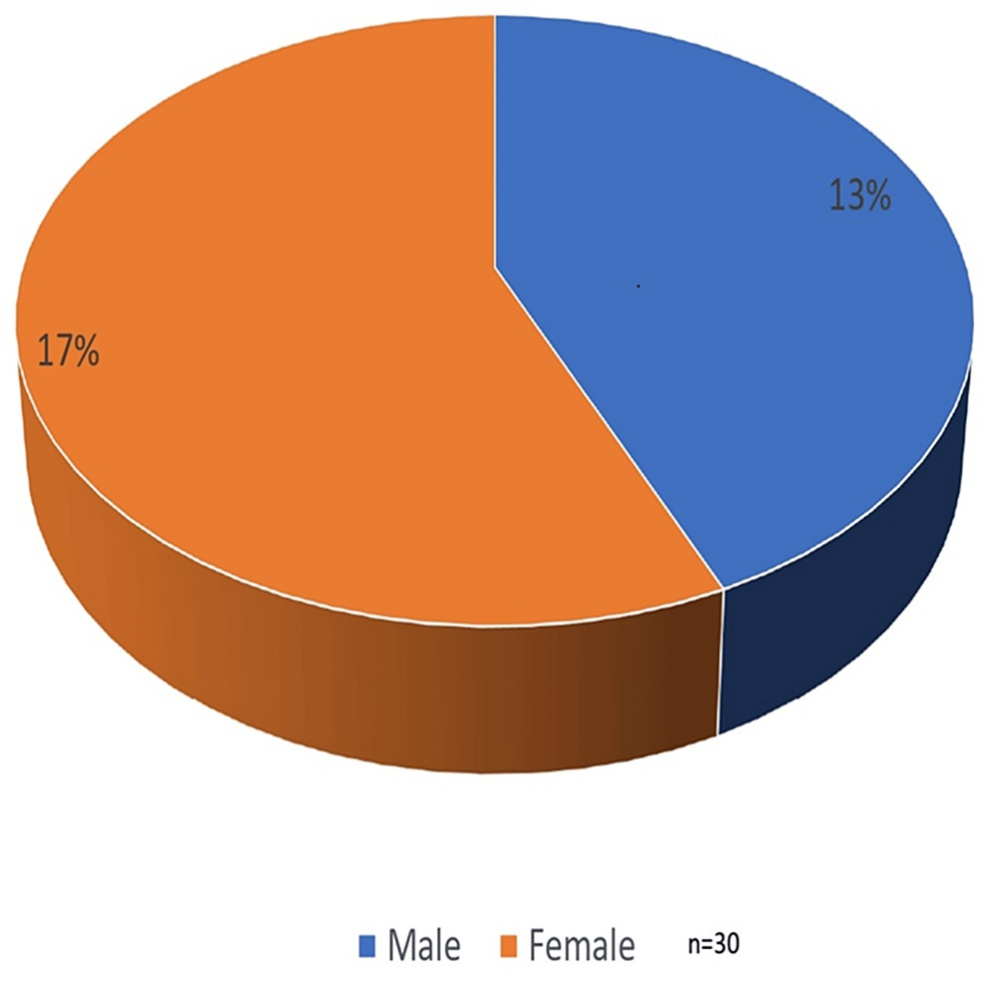
Gender-wise distribution

In this study, a total of 30 patients were included. The average age of the patients was 39, ranging from 22 years to 59 years, both men and women. The gender-wise distribution shown in Figure 1 shows that out of 30 patients, 13 (43.33%) were male and 17 were female (56.66%). The maximum illness duration varied for each subject from one month to several years. All cases were diagnosed as tuberculoid leprosy after their investigation and biopsy. In this study, we observe the involvement of both nerves in leprosy patients.

The anthropometric parameters of leprosy patients are depicted in Table 1. The average weight of the total cases was 59.6 kg, and the average height was 157.03 cm. Based on weight and size, the body mass index (BMI) was calculated. The average value of the BMI was 24.08 kg/cm^2^.

**Table 1:** Anthropometric parameters of the leprosy patients

Serial number	Parameter	Mean
1.	Weight (kg)	59.5
2.	Height (cm)	157.03
3.	BMI (kg/m^2^)	24.08

The latency and amplitude of the motor nerve were 6.61 ms and 3.89 mV, respectively. The ulnar nerve's average motor conduction velocity was 46.92 m/s, which is lower than the typical ulnar nerve conduction. The average values of latency and amplitude in sensory nerve conduction for the leprosy patients were 3.005 ms and 25.17 mV, respectively. The average value of SNCV was 38.00 m/s in leprosy patients. According to our investigation, there was an involvement of the sensory and motor nerves, which is depicted in Table 2.

**Table 2:** Motor and sensory nerve latency, amplitude, and conduction velocity in patients with leprosy

Nerve conduction velocity parameters	Latency	Amplitude	Conduction velocity
Motor nerve conduction velocity	6.61 ms	3.89 mV	46.92 m/s
Sensory nerve conduction velocity	3.005 ms	25.17 mV	38.00 m/s

## Discussion

Leprosy is one of the leading diseases due to *M. leprae* in the Indian population. Diagnosing leprosy is one of the most challenging tasks for doctors due to a lack of knowledge of patients who are not aware of the symptoms of the disease in the initial stages. Leprosy leads to severe neuropathy, and early diagnosis can help with the proper treatment and avoid the nerve involvement of patients. Early detection of leprosy can help to avoid neuropathy. Nerve conduction studies are one of the non-invasive techniques for the early diagnosis of leprosy.

Conduction studies have the advantage of quantitative findings from the research collection data and analysis reports, which will provide reference values for future studies and do not depend on the patient's cooperation or the observer's subjective experiences. They aid in the assessment of patients with peripheral neuropathy and disease progression, and therapeutic intervention monitoring. The only limitation is the expense of the investigation and the skills required to conduct it. The expenses are mainly for routine patients, as they would have to pay the charges for the NCV test, and the skills require more training for doctors to perform the NCV tests.

This study examined the involvement of motor and sensory nerves in leprosy patients using the ulnar nerve. It was found that in leprosy patients, the NCV of the ulnar nerve was reduced. This suggests maximum sensory-ulnar involvement in leprosy patients. Following the finding, sensory nerves are involved much earlier in patients with leprosy [18,19].

Furthermore, according to the result, it was observed that the sensory nerve involvement was early compared to the motor nerve involvement [20,21]. Anita et al. reported a similar observation that the ulnar nerve was affected more than the median nerve. It was in agreement with the study of Waffa Ramadan [22].

The sensory nerve is most affected in patients with leprosy. Though early identification depends on sensory nerve characteristics, neuropathy is the most common consequence in people with leprosy [23]. When comparing sensory fibers to motor fibers in the early stages of nerve damage, sensory fibers display a more significant number of decreased conduction velocities. Conversely, in the motor nerve fibers, the amplitude changes markedly [24].

In another study, it has been reported that motor nerve conduction was altered by 40% and sensory nerve conduction by 30% [25]. They observed the involvement of nerves in leprosy patients. In leprotic neuropathy, an electrophysiological investigation seems helpful for the prognosis and diagnosis of the disease. It indicates that skeletal muscle involvement in leprosy was secondary due to peripheral neuropathy. Further molecular-based and immunological studies are needed to assess the myographical involvement in leprosy patients.

Other variables of nerve conduction, including latency and amplitude, were observed in leprosy patients to provide a reference value. The latency was prolonged, and the amplitude was reduced in both motor and sensory nerves. Other studies suggested delayed nerve conduction velocity in leprosy patients due to C and A delta fibers' involvement in this nerve [26]. The amplitude and distal sensory nerve action potential were reported as more reliable parameters in leprosy neuropathy patients by Ramakrishnan et al. [27]. In another study, Mahajan et al. [28] found that their research affected motor nerves predominantly.

A similar finding in abnormal electrophysiological changes in sensory nerves correlates with hypoesthesia along the nerve root. Van Brakel and Khawas have suggested that all leprosy patients should undergo a nerve function examination at least during the first year of treatment [29]. Regular nerve function measurements were required to detect silent neuropathy early and avoid permanent nerve damage [30]. The limitation of our study was that the sample size was very small, so the results could not be precise. If this study is done at the multicentric level, it will have more significance.

## Conclusions

In our study, we observed decreased motor and sensory conductional velocities in the patient with leprosy. This data can be used as a reference value for future studies in leprosy patients for the diagnosis and management of the patients. Further, we can use normative data as a reference value for comparison with leprosy patients.
